# RSA, TSA and PyC hemi-prostheses: comparing indications and clinical outcomes using a second-generation modular short-stem shoulder prosthesis

**DOI:** 10.1007/s00402-020-03529-w

**Published:** 2020-10-06

**Authors:** Benjamin D. Kleim, Christina Garving, Ulrich H. Brunner

**Affiliations:** 1 Teaching Hospital of the Ludwig-Maximilians-University Munich, Munich, Germany; 2 Present Address: Department of Sports Orthopaedic Surgery, Klinikum rechts der Isar, Technical University Munich, Ismaningerstr 22, 81675 Munich, Germany

**Keywords:** Short stem, Shoulder arthroplasty, Pyrocarbon, Clinical, Hemiarthroplasty, Modular

## Abstract

**Introduction:**

The goal of this study was to provide an insight into the clinical results after modular short-stem shoulder arthroplasty for various indications.

**Materials and methods:**

A consecutive cohort study of 76 patients followed up for 23–55 (mean 31.4) months. 23 anatomical (TSA), 32 reverse (RSA) and 21 hemi-prostheses with a pyrocarbon head (PyC), using a modular short stem with proximal porous coating were implanted. Range of motion, pain and Constant score (CS) were recorded. Comparisons of pre- vs postoperative outcomes, between prosthesis types and indications, were made.

**Results:**

All prosthesis types brought about a significant improvement (*p* < 0.05) in all measured outcomes. TSA had a significantly higher increase in the CS than PyC and RSA (*p* = 0.002 and 0.003, respectively). TSA produced superior gains in all ROM compared with RSA (*p* < 0.02). RSA brought about significantly smaller improvements in internal rotation than TSA and PyC (*p* = 0.0001 and 0.008, respectively). TSA had greater pain relief than PyC (*p* = 0.02). TSA with Walch A glenoids seemed to improve more than type B in the CS. PyC patients with Walch B glenoids improved more than Walch A (*p* = 0.03). When implanted due to Osteoarthritis (OA), PyC had a comparable final outcome to TSA (*p* = 0.95), although the preoperatively worse TSA patients had a greater improvement in the CS (*p* = 0.026). The outcome of RSA did not differ between indications, but Walch A glenoids tended to improve more.

**Conclusions:**

Using a second-generation short-stem shoulder prostheses, TSA achieves the best clinical improvements overall, especially for OA with a Walch A glenoid. Despite refixation of the subscapularis tendon in all cases, RSA has inferior internal rotation than TSA and PyC, suggesting a mechanical limitation. OA, a Walch B glenoid and arthritis caused by instability seem to be ideal indications when considering PyC.

## Introduction

Shoulder arthroplasty is an increasingly common therapy for osteoarthritis, rheumatoid arthritis, cuff tear arthropathy, osteonecrosis as well as intra-articular fractures of the proximal humerus [1, 2].

Uncemented modular short-stemmed prostheses are still a relatively novel design type in shoulder prostheses and early results have been very positive with good function and low complication rates [3, 4]. A benefit is that one stem can be used in different configurations, as part of a hemi-prosthesis, RSA or anatomical TSA.

Hemi-prostheses are often considered for young patients with predominantly humeral disease, to avoid the complications of a glenoid replacement, subsequent bone loss and difficult revision surgery. However, a major problem when replacing only the humeral joint surface, traditionally with a cobalt-chrome head, is progressive glenoid wear and pain [5, 6]. Consequently, it has been found that TSA has a better outcome and more pain relief than hemiarthroplasty [7]. Pyrocarbon is a novel material thought to have biomechanical properties similar to cartilage and is therefore being used in hemi-prostheses in a hope to ameliorate this problem [8]. Clinical results achieved with this new material are yet scarce and to our knowledge no data exist where these are compared to total shoulder replacement.

A recent comparison of elderly patients receiving RSA or TSA for glenohumeral arthritis with an intact rotator cuff was unable to find a significant difference in outcomes [9]. However, other studies have shown TSA to be superior in external rotation [10] and in internal rotation when compared in patients who had contralateral implantation of both RSA and TSA [11].

For cuff tear arthropathy there is a consensus that joint replacement should be performed with RSA. However, for primary osteoarthritis a TSA, RSA or hemi-prosthesis may be used. The choice of which type to implant is based on the patient age, function and disease morphology. The morphology of glenoid wear, as described by Walch and later modified by Bercik [12], has been shown to impact on the outcomes after shoulder arthroplasty. Outcomes of hemi-prostheses were found to be adversely affected by eccentric posterior wear [13]. This trend has also been described for outcomes after TSA, although these still had better results than hemi-prostheses in patients with Walch B2 glenoids [14]. For this reason a trend has emerged to opt for RSA in cases with excessive posterior glenoid wear [15].

The aim of this study was to investigate and compare the clinical outcomes of this second-generation short-stem modular shoulder prosthesis, when used in its different forms (TSA, RSA and PyC) and for different indications.

## Materials and methods

### Patient population and study design

In this single-centre cohort study, all 103 patients who consecutively underwent shoulder arthroplasty, using a curved titanium short-stem uncemented modular prostheses with a proximal porous coating (Aequalis Ascend Flex™, Wright Medical, Bloomington, USA), between May 2013 and June 2015 at Agatharied hospital, were invited for follow-up at regular intervals. All the operations were carried out by one of two senior surgeons.

Preoperatively the glenoid retroversion was calculated relative to the Friedman line [16], the inclination according to the Maurer angle [17] using X-rays and CT. The prostheses were implanted in three forms: Hemiarthroplasty using a pyrocarbon head (PyC), anatomic total shoulder prosthesis (TSA) and reversed shoulder prosthesis (RSA). Patients were offered prosthesis types best suited to their pathology: Patients with primary osteoarthritis, intact rotator cuffs, a glenoid retroversion of < 10° and a posterior subluxation of the humeral head of < 80% underwent anatomical TSA; patients with little or no glenoid pathology, an intact rotator cuff and younger age were offered a PyC hemiprosthesis; lastly patients with rotator cuff pathology, glenohumeral subluxation of > 80% or a glenoid retroversion of 10° or more were treated with RSA. If the retroversion was > 10°, this was corrected with the use of autologous wedge-shaped cancellous bone grafting (wedged BIO-RSA) under the base plate (4 cases). Furthermore, in 2 cases with extreme glenoid wear, BIO-RSA was employed to lateralize the base plate. The subscapularis tendon was repaired in all cases transosseously in double-row technique.

10 Patients were excluded from this study: 1 with a hemiprosthesis with a titanium head (in place of the pyrocarbon due to nickel allergy) and 9 with complications as outlined in the results below, leaving a potential study group of 93. Data from preoperative examinations as well as at most recent follow-up were gathered and analysed. The glenoid morphology was described according to the modified Walch classification [12] from the preoperative CT. We had follow-up data for 76 of the eligible 93 patients (82%). Patients were lost to follow-up for reasons, such as old age and frailty, death (unrelated to the operation or prosthesis), missing data or refusal of follow-up examinations.

### Clinical evaluation

Preoperatively and during follow-up appointments clinical outcomes, such as range of movement (ROM) and pain on the visual analogue scale (VAS), as well as the validated Constant Score (CS) [18], were recorded. This and further patient information including the demographics, diagnosis and operations were gathered from the patient records. To quantify internal rotation, this was scored as shown in Table 1.

**Table 1:** Scoring system for internal rotation

Internal rotation score	Level reached with the back of the hand
0	Thigh
1	Gluteal
2	Iliosacral joint
3	Lumbar spine
4	Thoracic spine
5	Scapula

### Statistics

The statistics software SPSS V25.0 (IBM) was used. To assess the significance of changes in pre- and post-operative outcomes, the paired *t* test was calculated; to compare the delta values (difference between pre- and postoperative) between implant types, the *t* test was employed. In each case the significance threshold was set at *p* < 0.05.

## Results

### Patient demographics

The patient demographics and diagnoses leading to surgery of the study group of 76 patients are shown in Table 2. With the exception of the PyC subgroup, the cohort was made up of more women than men. The PyC contingent was younger and male dominated. The average age was the highest in the RSA subgroup.

**Table 2:** Patient demographics and preoperative diagnoses

	Total	TSA	RSA	PyC
Number	76	23	32	21
Age	68.5 (22–84)	70.0 (58–84)	74.1 (65–84)	58.3 (22–84)
Sex	45 female	15 female	26 female	4 female
Follow-up (months)	31.4 (23–55)	31.6 (23–51)	34.3 (23–55)	26.7 (23–38)
Primary osteoarthritis	44	23	6	14
Cuff tear arthropathy	24	0	25	0
Irreparable rotator cuff tear	1	0	1	0
Fracture sequelae	1	0	0	1
Avascular necrosis	3	0	0	3
Arthritis resulting from instability	3	0	0	3

Table 3 shows the glenoid morphologies of the patients prior to surgery. TSA patients had mostly A2 glenoids, but also B1 and B2 wear patterns. RSA also had predominantly A type glenoids, but also B and D morphologies. The PyC subgroup contained patients with mixed glenoid types.

**Table 3:** Preoperative glenoid morphology according to the modified Walch classification [18]

Glenoid type (Walch)	Total	TSA	RSA	PyC Hemi
A1	14	0	9	5
A2	28	13	11	4
B1	19	6	7	6
B2	11	4	2	5
B3	0	0	0	0
C	0	0	0	0
D	4	0	3	1

### Pre- vs postoperative clinical outcomes

The clinical outcomes pre- vs postoperative are displayed in Table 4. All patient groups improved significantly (*p* < 0.05) in all outcomes measured. TSA patients had, on average, the lowest preoperative and the highest postoperative CS. The PyC patients had the highest preoperative CS. All patient groups benefited from pain reduction, reducing from VAS 6.6 pre-change to preoperatively down to 1.0 postoperatively. Abduction increased from 88° to 129°, forward flexion from 93° to 137°. Internal rotation increased from reaching the gluteal area before, to placing the back of the hand on the lumbar spine after the operation. On average, patients also benefited from more external rotation, 21° preoperatively and 40° postoperatively.

**Table 4:** Pre- and postoperative outcomes for the measured variables for the cohort and subgroups

Outcome	Total pre-OP	Total post-OP	TSA pre-OP	TSA post-OP	RSA pre-OP	RSA post-OP	PyC pre-OP	PyC post-OP
CS	38.2	78.3	34.5	84.0	36.9	72.9	44.2	80.3
Pain VAS	6.6	1.0	6.7	0.37	6.8	1.1	6.4	1.5
Abduction°	88.2	129.4	84.3	138.3	88.1	121.7	92.6	131.4
Flexion°	92.5	137.1	87.6	144.3	91.4	128.0	99.5	143.1
Internal rotation (score)	1.4	2.6	1.0	3.1	1.7	2.3	1.2	2.7
External rotation°	21.1	39.6	6.1	40.0	19.8	36.3	17.6	44.3

All comparisons between pre- and postoperative values were statistically significant (*p* < 0.05)

### Comparison between implant types

**Fig. 1:**
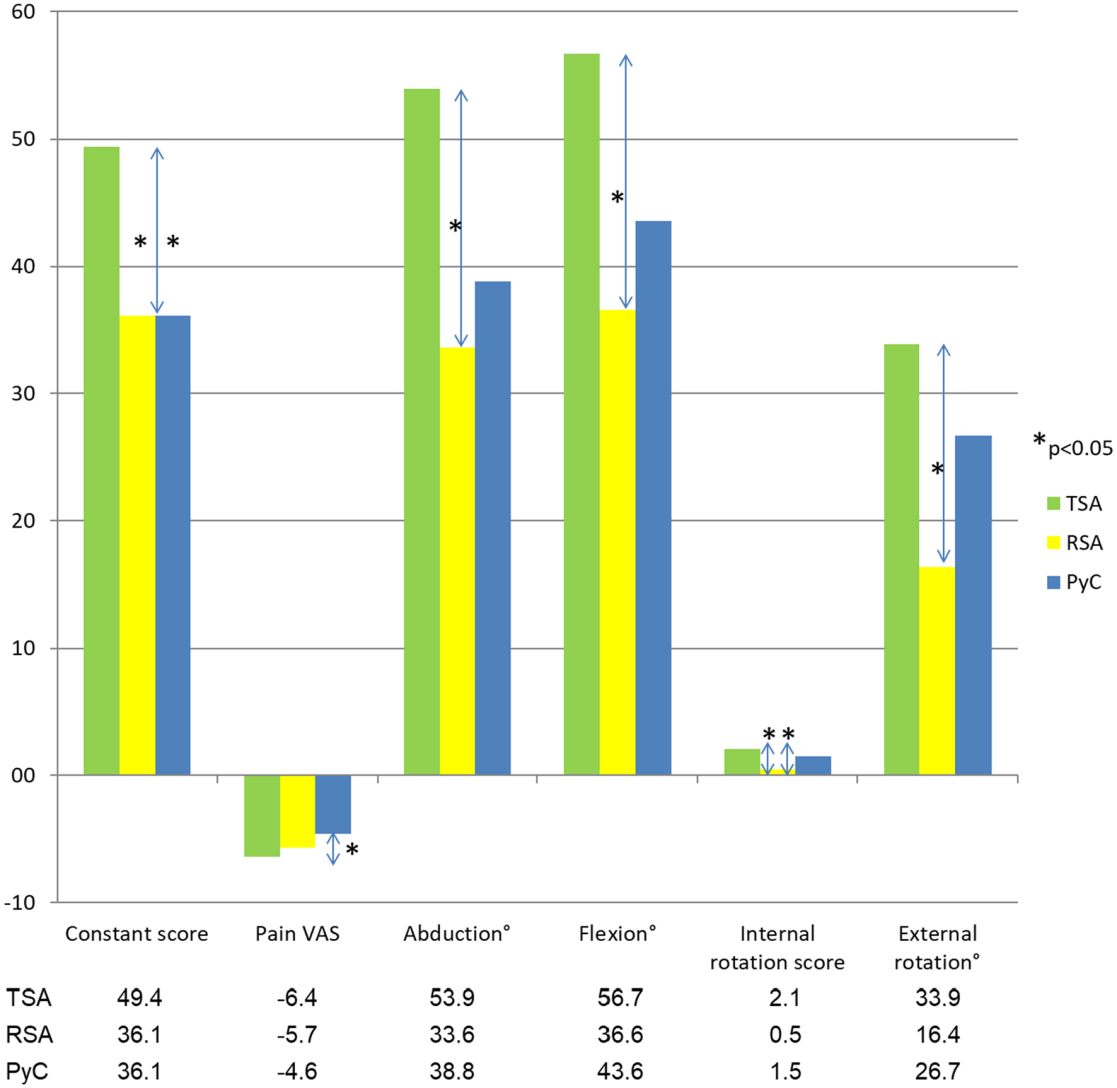
Delta (postoperative minus preoperative) values for the measured outcomes compared between the implant types. Statistically significant (*p* < 0.05) differences are identified with a *

To reduce the confounding effect of the demographical differences in the subgroups, delta values (difference between pre- and postoperative) were compared between prosthesis types (Fig. 1).

Notably, anatomical total shoulder replacements had a significantly higher increase in the CS than the other two types. They also brought about more pain alleviation than the pyrocarbon hemiarthroplasties. In abduction and flexion the RSA had less improvement than the TSA. Both the TSA and the PyC gained more internal rotation than the RSA. Additionally, the TSA had larger improvements in external rotation than the RSA. All other comparisons between the implants were not statistically significant. Nevertheless, the trend emerged that TSA was superior to the other two in all aspects and PyC bettered the RSA in everything but pain reduction.

Given that glenoid morphology is an important predictor of outcome, we investigated the performance of the different prostheses when used in patients of varying glenoid types (see Table 5). Interestingly the final outcome of TSA did not differ between A and B glenoids. However, the improvement achieved with TSA seems to be greater in A than in B glenoid patients, although this was not statistically significant (*p* = 0.085). Conversely, using PyC, patients with B glenoids improved significantly more than those with A glenoids (*p* = 0.034), again achieving the same final outcome. The one patient with a D glenoid in PyC had a poor preoperative function and improved less than A and B patients. When comparing the result with PyC vs TSA in arthritis with B type glenoids, little difference is seen in the outcome or the function gained. In patients with type A glenoids, however, a significantly greater improvement was observed using TSA compared with PyC (*p* < 0.0001). Both subgroups reach a similar postoperative CS.

**Table 5:** Comparison of mean constant score by prosthesis type and glenoid morphology

Glenoid type (modified Walch)	Prosthesis type		
	TSA	RSA	PyC
A			
Preoperative CS	28.9	30.4	52.7
Postoperative CS	83.5	71.5	81.7
Delta CS	53.7	41.1	29.0
B			
Preoperative CS	40.6	47.8	39.5
Postoperative CS	84.5	76.6	82.8
Delta CS	43.9	28.8	43.3
D			
Preoperative CS	–	49.7	18.0
Postoperative CS	–	74.0	39.0
Delta CS	–	24.3	21.0

In the RSA subgroup patients with Walch A glenoids appeared to have the greatest improvement, but this was not significant (A vs B *p* = 0.091; A vs D *p* = 0.15). The postoperative CS was comparable between all three glenoid types.

Table 6 differentiates the results achieved when applying the different prosthesis types for the various diagnoses. The comparison for TSA and PyC hemi-prostheses in osteoarthritic shoulders revealed a significantly greater improvement in the CS of TSA patients (*p* = 0.026), which had a lower preoperative function. However, there is no difference in the final outcome of the PyC and TSA subgroups (*p* = 0.95). When comparing RSA with TSA in osteoarthritis patients, TSA has a significantly greater increase in the CS (*p* = 0.011) and a better postoperative CS (*p* = 0.002). When comparing the results achieved for OA, there was no significant difference in the delta CS using PyC or RSA (*p* = 0.43). Although PyC seemed to have a better final result, this was not statistically significant (*p* = 0.058).

**Table 6:** Comparison of mean CS achieved using the different prostheses for the various diagnoses

Diagnosis	Prosthesis type		
	TSA	RSA	PyC
Primary osteoarthritis			
Preoperative CS	34.5	42.5	45.6
Postoperative CS	84.0	74.7	83.8
Delta CS	49.5	32.2	38.2
Cuff tear arthropathy			
Preoperative CS	–	34.8	–
Postoperative CS	–	72.4	–
Delta CS	–	37.6	–
Irreparable rotator cuff tear			
Preoperative CS	–	53	–
Postoperative CS	–	75	–
Delta CS	–	22	–
Fracture sequelae			
Preoperative CS	–	–	50
Postoperative CS	–	–	61.5
Delta CS	–	–	11.5
Avascular necrosis			
Preoperative CS	–	–	41.7
Postoperative CS	–	–	68
Delta CS	–	–	26.3
Arthritis resulting from instability			
Preoperative CS	–	–	38.3
Postoperative CS	–	–	82.3
Delta CS	–	–	44

Comparing the results for different indications within the PyC subgroup, this showed no significant differences in the delta scores or postoperative CS between any diagnoses (*p* > 0.05). However, patients operated for OA and arthritis resulting from instability seemed to have a greater benefit and a better postoperative outcome than those with avascular necrosis and fracture sequelae. Although not statistically significant (or measurable using a *t* test), the patient with arthritis resulting from a fracture had the worst postoperative CS and the least improvement compared to the mean values of the other diagnoses.

In the RSA subgroup patients operated for CTA did not improve significantly differently in the CS (*p* = 0.53), nor did they reach a different outcome according to the CS (*p* = 0.72), when compared with patients with OA. The patient with the irreparable rotator cuff tear, although not statistically testable, also appeared to have an outcome comparable to the other RSA patients.

### Complications

In the study group of 76 patients, 2 developed stress fractures: 1 of the scapular spine, which was successfully operated with an ORIF; one of the acromion, which was treated conservatively. 2 patients were revised with evacuations of postoperative haematomas. There were 2 cases of postoperative anaemia requiring a blood transfusion (2.7%) and 4 cases had neurological deficits postoperatively which resolved spontaneously in the months following surgery.

### Complications leading to exclusion

9 patients had to be excluded as a result of complications which were recorded in the follow-up of the initial patient cohort of 103 shoulder prostheses, as they were no longer deemed comparable to the rest of the cohort: 4 patients were found to have a low-grade infection (3.88%) and had to be revised. 2 of these were RSA patients, one of whom was 88 years old, the other was a psoriasis vulgaris patient under methotrexate therapy. TSA and PyC had one case of infection each; in the case of the PyC this was a patient who had had a previous operation due to a tubercular fracture. 2 of the 4 infections were caused by Cutibacterium acnes (formerly known as Propionibacterium acnes). One patient with a TSA developed a rotator cuff tear and was converted to RSA. There were 2 patients with periprosthetic fractures of the humerus (1.94%), one of whom was treated at another hospital. The other fell and fractured twice, she was treated conservatively the first time, the second time she was operated with a single cerclage with a good outcome. One RSA patient with extremely osteoporotic bone suffered a bony dislocation of the glenoid component, despite the use of a long peg base plate and 4 screws. This was revised, the glenoid component removed and as a salvage operation a modular exchange to place an anatomical head on the existing shaft was performed. 1 patient underwent a cervical spine operation in the months after her shoulder prosthesis and suffered a neurological deficit ipsilaterally, involving the deltoid, as a complication of this. This resulted in recurrent dislocations of the shoulder prosthesis and a stress fracture of the scapula spine of the operated side, leading to exclusion.

## Discussion

### Key results

This patient cohort showed significant improvements in all measured ROM, pain and the CS compared to preoperative values, using a modular short-stem prosthesis with a proximal porous coating 2–4 years postoperatively. This correlates with previous findings using first-generation short-stemmed shoulder prostheses [3, 4, 19, 20]. The overall very positive clinical outcomes achieved with these shoulder prostheses are also, at least, comparable to those described with the use of standard-stemmed prostheses with diaphyseal anchoring [21, 22].

The modular design of this prosthesis has the advantage of individualized assembly to recreate the anatomy of the proximal Humerus and balance tension of the soft tissues. Eccentric head/tray (depending on anatomical or inverse design) components allow the surgeon to adjust the position of the head/tray by turning it to the ideal position before fixation. In this way ideal coverage without overlap and adjustments of soft tissue tension can be made regardless of the stem position. This is of particular importance in the anatomical prosthesis and may in part have contributed to the successful clinical outcomes.

The hemiprosthesis which was used in this cohort uniformly utilized a novel pyrocarbon head. This material is thought to have a biomechanical profile close to that of cartilage and therefore is hoped to reduce the problem of glenoid wear and pain which complicate traditional hemiarthroplasties [8]. Preliminary results after implantation of these have been encouraging, except in patients with a diagnosis of fracture sequelae (osteonecrosis or secondary osteoarthritis), in a study containing some of the patients from our cohort [23]. Our results equally are encouraging, with improvements in all areas of clinical function. With regard to results in the use for fracture sequelae, we had one patient operated with PyC for this indication, which yielded a poorer result than all other indications, adding weight to the conclusion drawn by Garret et al. [23].

The RSA used in this cohort has a neck shaft angle (NSA) of 145°. This is an intermediate value between the 135° and 155° which inverse prostheses also commonly have. Mechanical studies have shown that a steeper NSA causes earlier impingement in abduction, but reduces glenoid notching and increases joint stability [24]. Computerized models have found a lower NSA (135°) to allow a greater ROM in all motions except abduction [25]. This was also true of internal rotation, although the effect of the lower NSA on internal rotation was negated when the glenosphere had been lateralized. Our cohort demonstrated a successful clinical outcome with this modular tray with a NSA of 145° in terms of the ROM, with no dislocations, suggesting this may be a good compromise between mobility and stability.

The comparison between the different types of this modular prosthesis was interesting, as it showed several differences in their functional characteristics. Overall, though not always statistically significant, the TSA achieved the best outcomes in all measured variables. Kiet et al. have previously described the outcomes of RSA and TSA to be similar, with only better rotation in the TSA group [10]. They, however, did not compare the delta values of the measured parameters. Contradictory to our findings, Flurin et al. described higher outcome scores in TSA but comparatively greater gains in RSA patients [26]. They had implanted the Equinoxe shoulder platform system (Exactech Inc., Florida). Differences in results compared to our study may be due to the implant or differences in the patient cohorts. Trends emerged that patients with PyC heads generally had greater improvements in the ROM than RSA, whilst RSA brought about more pain relief than PyC. However, the only statistically significant difference between the PyC and the RSA was that PyC had greater improvement in internal rotation. This is mirrored by better internal rotation in TSA compared to RSA patients and is a reproducible finding [10, 27]. As we reconstructed the subscapularis tendon in all prosthesis types, this phenomenon may likely be explained as being a result of a mechanical restriction of the RSAs semi-constrained design.

### Comparison between glenoid types

When comparing glenoid types, one important finding was that the final outcome did not differ between TSA patients which had type A or B glenoids. It appears, though, that there may be a greater increase in the CS when operating patients with a centred type A situation. This may be because A glenoids have a purely arthritic problem, which can be solved by replacing the glenoid surface, whereas in the B type situation a soft tissue imbalance complicates the disease. PyC patients with B type glenoids improved more than those with A glenoids, reaching a similar end result. This implies that the presence of a B glenoid does not contraindicate the use of a PyC hemiprosthesis and rather laments that it may be a good indication. It seems that the additional benefit the A types have over B types in TSA is lost in PyC, as the glenoid is not replaced. Strengthening to this is that patients with B glenoids had comparable outcomes when treated with TSA or PyC, whereas type A glenoids improved significantly more when treated with TSA. This does not entirely fit with the findings of Iannotti, who found that both TSA and hemi-prostheses had worse outcomes in a Walch B2 setting, where TSA was still the better choice [14]. This may be because we grouped all B type glenoids together (roughly equal numbers of B1 and B2), whereas they only looked specifically at B2. Also, they used standard cobalt-chrome heads for their hemi-prostheses, so it may be that the new pyrocarbon heads have different clinical properties to these. Perhaps in a type B1 situation with little glenoid wear, the natural glenoid with its labrum is superior to a prosthetic glenoid, giving rise to more clinical improvement when the corresponding arthritic humeral articular surface is replaced. Furthermore, it may be that in a B2 setting with biconcave posterior wear PyC is more effective than standard cobalt-chrome prostheses.

In RSA it appears as if patients with type A glenoids may have the most benefit from the operation. This may be because these are the ones that have cranialized more, rather than posteriorizing and therefore benefit from the distalization of the RSA more. The final outcome appears similar between all glenoid types, including type D, however.

### Comparison between Diagnoses

When comparing outcomes between the different types of prostheses in terms of results achieved for osteoarthritis, these mirrored what we found overall when comparing RSA to the other two types. The comparison between TSA and PyC showed a greater improvement in TSA patients with OA, as was the case in the comparison of the entire subgroups for the prostheses. However, the final outcome of the PyC, when only considering OA patients, now matched that of the TSA patients much more closely. In TSA therefore, older patients with worse preoperative function improve more than the on average younger PyC patients. Nevertheless, the function achieved after 2 years when using PyC for OA is equal to that of the TSA.

To allow some insight into what a good indication for the novel PyC hemiprostheses may be, it was interesting to try and compare the results achieved for the various diagnoses. Unfortunately, the group sizes were too small to show any significant differences in this regard. It can be said, though, that there is a trend that OA and arthritis resulting from instability may be the best indications for implanting a PyC hemiarthroplasty.

The improvements and outcomes achieved using RSA seem to be the same irrespective of the preoperative diagnosis. This may be because it has inherent stability due to its semi-constrained design, allows recruitment of the deltoid muscle to substitute rotator cuff function and is limited more by mechanical impingement.

### Complications

The infection rate of 3.88% documented in our cohort is comparative to findings of a meta-analysis by Zumstein et al. who found an infection rate of 3.8% in RSA patients [28]. Rates of 1–3.9% have been described for TSA as well [29], whilst it is thought that rates in RSA patients are higher due to haematoma formation in the increased dead space [30]. This is in line with our results. Differences in the reported infection rates are likely to be a result of follow-up and diagnostic differences. Rates of low-grade infections are often likely to be higher than reported as they are difficult to distinguish from aseptic failure [31]. Cutibacterium acnes (formerly Propionibacterium acnes), which made up half of our 4 cases, has been reported to be present in 18–60% of infections [30]. It is found in the deep tissues around the shoulder, more commonly in men, when using the deltopectoral approach and it has been found in the joint fluid of 42% of patients undergoing primary shoulder arthroplasty and more often still in revision surgery [30, 32, 33].

The rate of periprosthetic humeral fractures in our cohort (1.94%) correlates with 1.6–2.4% described in the literature for standard shaft prostheses [34]. However, we were able to treat a patient with a recurrent periprosthetic fracture successfully with as little as a single cerclage. This may indicate simpler treatment options for periprosthetic fractures around short-stem prostheses, but clearly more data are required to draw any conclusions regarding this. Regarding the two cases of scapula spine and 1 acromion fracture in the potential cohort of 103 patients, it should be noted that these all occurred in RSA patients. This is unlikely to be a coincidence as reverse shoulder prostheses put a lot of stress on the delta muscle which causes tension and can lead to stress fractures of the scapula. This affect may be exacerbated in this model due to the additional lateralization and distalization brought about by the onlay design of the modular tray.

### Limitations and generalizability

The main limitation of this study is the retrospective design and tailored patient selection for the different subgroups, leading to selection bias. The resulting differences in diagnostic indications for arthroplasty and patient demographics in the subgroups limit the extent to which interventions can be reliably compared. Furthermore, the sample size, although when compared with other similar studies in the literature is large, may have inhibited the emergence of further statistical significances in the comparison between the prosthesis’s subtypes. The follow-up rate of 82%, which for a mid-term cohort study is satisfactory, could to some extent jeopardize the generalizability of the study. Furthermore, whilst using a single-centre study design increases reliability in a scientific method, it also reduces the extent to which results can be extrapolated to other settings. This must be taken into account when interpreting results. The treatment of this cohort took place before we adopted the use of 3-dimensional planning, so it is not known if and how much correction of version and/or inclination was achieved. Another limitation is that although the subscapularis tendon was repaired in all cases, we did not carry out any controls of the success of this repair.

## Conclusions

This study provides an insight into the clinical properties of the different forms in which modular short-stem shoulder prostheses can be implanted and their results for various indications.

When implanted for the diagnoses for which each were conceptualized, it can be said that TSA patients can expect the greatest clinical improvement postoperatively compared to RSA and PyC. PyC patients seem to have bigger improvements in ROM than RSA patients but may subjectively have less pain reduction. Interestingly, despite refixation of the subscapularis tendon in all prosthesis types, RSA had less improvements in internal rotation than the other two prosthesis types, which suggests a mechanical restriction of RSA.

When comparing the clinical success of these prostheses when used for different glenoid types according to the modified Walch classification, we can surmise that TSA has the same outcome regardless of A or B glenoid type, but possibly with more improvement for type A glenoids. Novel PyC hemiprostheses are a good indication for patients with type A and type B glenoids, with equal clinical outcomes and a greater improvement in B glenoids. In fact, type B glenoids were treated just as successfully with PyC as with TSA.

If OA is the indication for arthroplasty, TSA and PyC achieve a comparable clinical result 2–4 years postoperatively, although the preoperatively worse TSA patients have more improvement. It can be said that if glenoid replacement is to be avoided, for example due to young age, that OA patients or those with arthritis resulting from instability and especially in the presence of a type B glenoid seem to be good candidates for treatment with PyC.

The diagnosis for which RSA is implanted does not seem to greatly affect the outcome, but those with a Walch A glenoid may improve more.
